# Experimental Findings and Clinical-Pathologic Correlation of Radiofrequency Catheter Ablation at the Left Ventricle Para-Hisian Region

**DOI:** 10.3389/fcvm.2021.793903

**Published:** 2022-01-27

**Authors:** Zuyi Fu, Zili Liao, Jinlin Zhang, Xianzhang Zhan, Weidong Lin, Fang Zhou Liu, Xi Su, Hai Deng, Xianhong Fang, Hongtao Liao, Hongyue Wang, Shulin Wu, Yumei Xue, Feifan Ouyang

**Affiliations:** ^1^Department of Cardiology, Guangdong Provincial People's Hospital, Guangdong Academy of Medical Sciences, Guangzhou, China; ^2^Department of Cardiology, Wuhan Asian Heart Hospital, Wuhan, China; ^3^Department of Pathology, National Center for Cardiovascular Diseases, Fuwai Hospital, Chinese Academy of Medical Sciences and Peking Union Medical College, Beijing, China; ^4^Southern Medical University, Guangzhou, China; ^5^Universitäres Herz- und Gefäßzentrum, University Hospital Eppendorf, Hamburg, Germany

**Keywords:** his bundle, 3-D anatomical, catheter ablation, pathology, pacing

## Abstract

**Background:**

Catheter ablation target at the site with large His activation in the left ventricle poses a high risk of atrioventricular (AV) block. We aimed to identify far-field (FF) and near-field (NF) His activation at left upper septum (LUS).

**Methods:**

Three-D mapping of the aortic root and left ventricle was performed in 12 dogs. Two sites located at either the base or apex of the triangle interposed between the hinges of the the noncornary coronary cusp (NCC) - right coronary cusp (RCC) were chosen for a single radiofrequency (RF) application. Bipolar and unipolar pacing with different outputs at both sites was attempted to discern NF and FF His activation.

**Results:**

The sites chosen for NF and FF ablation were located at the base and apex of the triangle, which were 8.03 ± 1.18 mm (group 1) and 3.42 ± 0.61 mm (group 2) away from the RCC-NCC junction. Lower A/V ratios were found in group 1. Pacing could not differentiate NF from FF His activation. In group 1, ablation resulted in III degree AV block in all 6 dogs, whereas neither PR prolongation nor AV block occurred in group 2. Pathologic examination of group 1 showed complete/partial necrosis of the His bundle (HB) and left bundle branch in all 6 dogs. In group 2, no necrosis of the HB was seen in the 6/6 dogs.

**Conclusion:**

Anatomical localization in the triangle of RCC-NCC junction can help differentiate NF from FF His activation.

## Introduction

Understanding the anatomy and identification of near-field (NF) and far-field (FF) His activation of the Para-Hisian (PH) area is critical for catheter ablation of PH arrhythmias (1, 2). Catheter ablation targeted at the site with a large His activation in the left ventricle (LV) can lead to a high risk of atrioventricular (AV) block, often abortion of procedures or targeting of neighboring sites associated with high recurrence rates. Our previous studies demonstrated that (1) the His potential with either FF or NF activation is widely distributed in the right PH region, and the NF or FF His activation can be identified using systematic pacing maneuvers; (2) ablation at the site with FF His activation can be safely performed without injuring the AV conduction system (AVCS) (1, 2). Our strategy is widely used to guide radiofrequency (RF) ablation of right-sided PH arrythmias including PH accessory pathways and ventricular arrhythmias. However, it was unknown whether this strategy can be extrapolated to left upper septal ablation. The aim of this study was to investigate the distribution of His activation at the left upper septum (LUS) below the noncornary coronary leaflet (NCL)- right coronary leaflet (RCL) junction, to identify FF and NF His activation within the region using pacing techniques, to deliver a single RF ablation at the apex or base of the triangle interposed between the hinges of the NCL and RCL of the aortic root and finally to correlate with clinical and pathological characteristics of AVCS injury in the canine model. We include a case of successful ablation of a left PH accessory pathway in a patient with previous failed ablation attempts.

## Method

### Electrophysiological Procedure

Twelve mongrel dogs with a weight of 27.1 ± 3.5 kg (range 22.0–33.5 kg) were anesthetized and intubated with an endotracheal tube and mechanically ventilated as previously reported (1). High frequency/low tidal volume with a volume of 200 ml and a frequency of 30 breaths per minute (bpm) was used during mapping and ablation in the left PH region. Standard limb and V1, V3 and V5 leads of the surface ECG were continuously monitored and documented throughout the entire procedure. Subsequently, two 6F catheters were placed into the coronary sinus (CS) and at the right ventricle under fluoroscopic guidance *via* the right jugular vein and left femoral vein, respectively. All bipolar intracardiac electrograms were filtered at 30–500 Hz and recorded using a LABSYSTEM^TM^ EP recording system (Bard, Boston-Scientific, MA, USA). Programmed atrial stimulation from the CS was performed to determine the antegrade conduction over the AVCS.

### Three-Dimensional Electro-Anatomical Map

**Figure 1:**
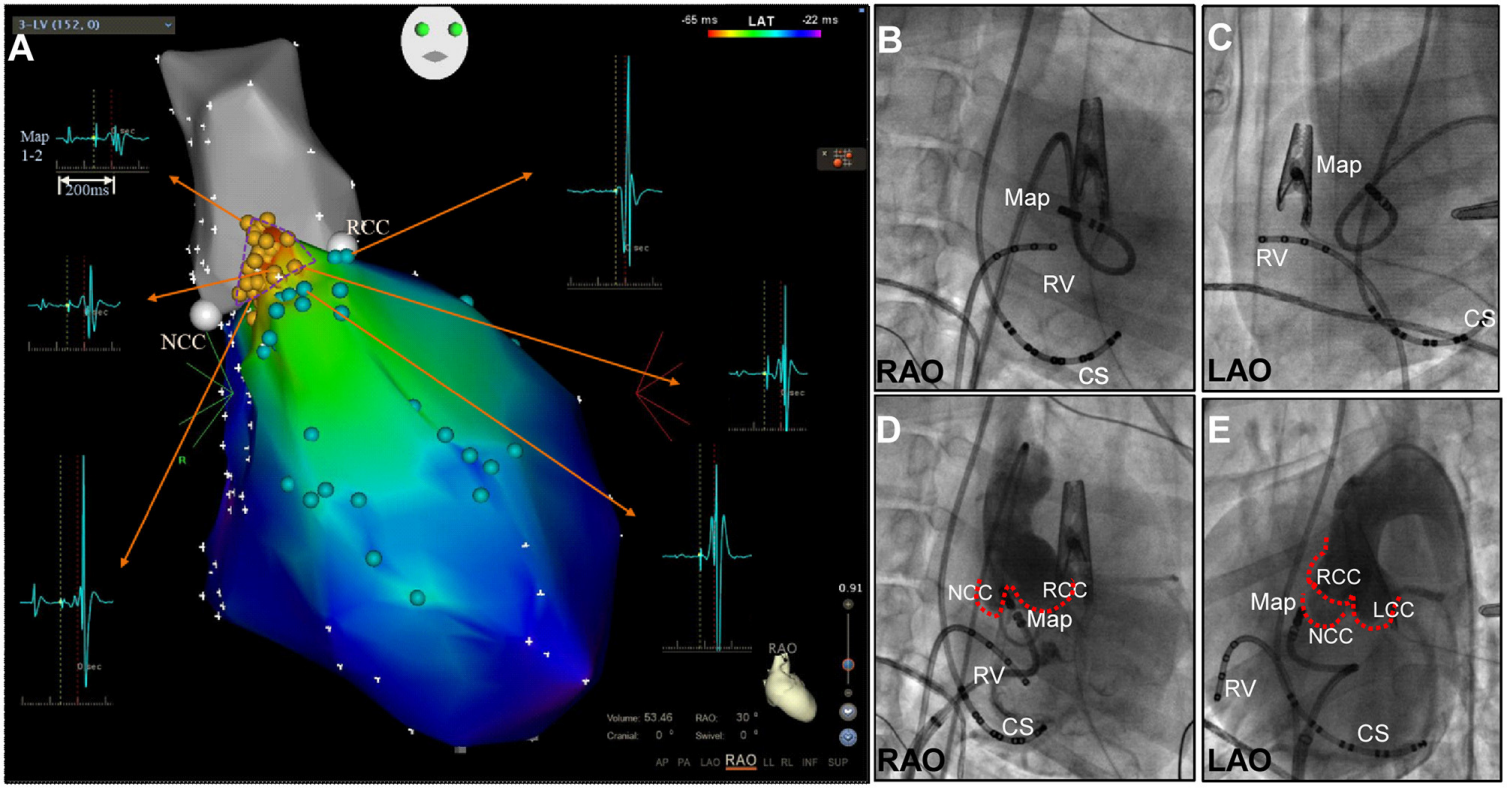
**(A)** Three-dimensional mapping of the aortic root (grey) and upper septal region in the left ventricle in right anterior oblique (RAO) view. A small triangular area with His activation was found between the NCC and RCC. His activation (yellow points), bundle branch potential and branches (light green points). **(B-E)** Fluoroscopic right anterior oblique (RAO) and left anterior oblique (LAO) views demonstrating mapping at the apex of the NCC-RCC junction via the retrograde transaortic approach **(B,C)** and transseptal approach with the reverse S-curve **(D,E)**. CS, coronary sinus; LCC, left coronary cusp; Map, mapping catheter; NCC, non-coronary cusp; RCC, right coronary cusp; RV, right ventricle.

3-D electro-anatomical mapping (CARTO, Biosense Webster, CA, USA) of the LV was performed as previously reported (3). In brief, mapping was performed using a 7.5F D-curve catheter with a 3.5-mm irrigated-tip electrode (Navistar Thermo-Cool; Biosense Webster, Diamond Bar, CA). Initially, the aortic root was mapped via a retrograde approach to identify the noncornary coronary cusp (NCC), right coronary cusp (RCC) and left coronary sinuses (LCC), to highlight the anatomical location of LUS in the LV. Subsequently, high-density mapping of the LV was performed at the LUS including identification of the HB, left bundle branch(LBB) and proximal branch of the LBB during sinus rhythm (SR) (Figure 1). The transseptal approach with an 8.5F SL1 long sheath (St Jude, Minneapolis, MN, USA) was attempted to reach the upper septal region with a reverse S curve of the ablation catheter (Figures 1D,E) (3). Activation mapping was performed as previously reported (1). The site of the His potential in the LV, which, defined as the potential with the longest interval from the Purkinje potential to the ventricular activation as well as an atrial activation, was tagged on the 3-D map (Figure 1A).

### Pacing Protocol at the Region With His Activation

**Figure 2:**
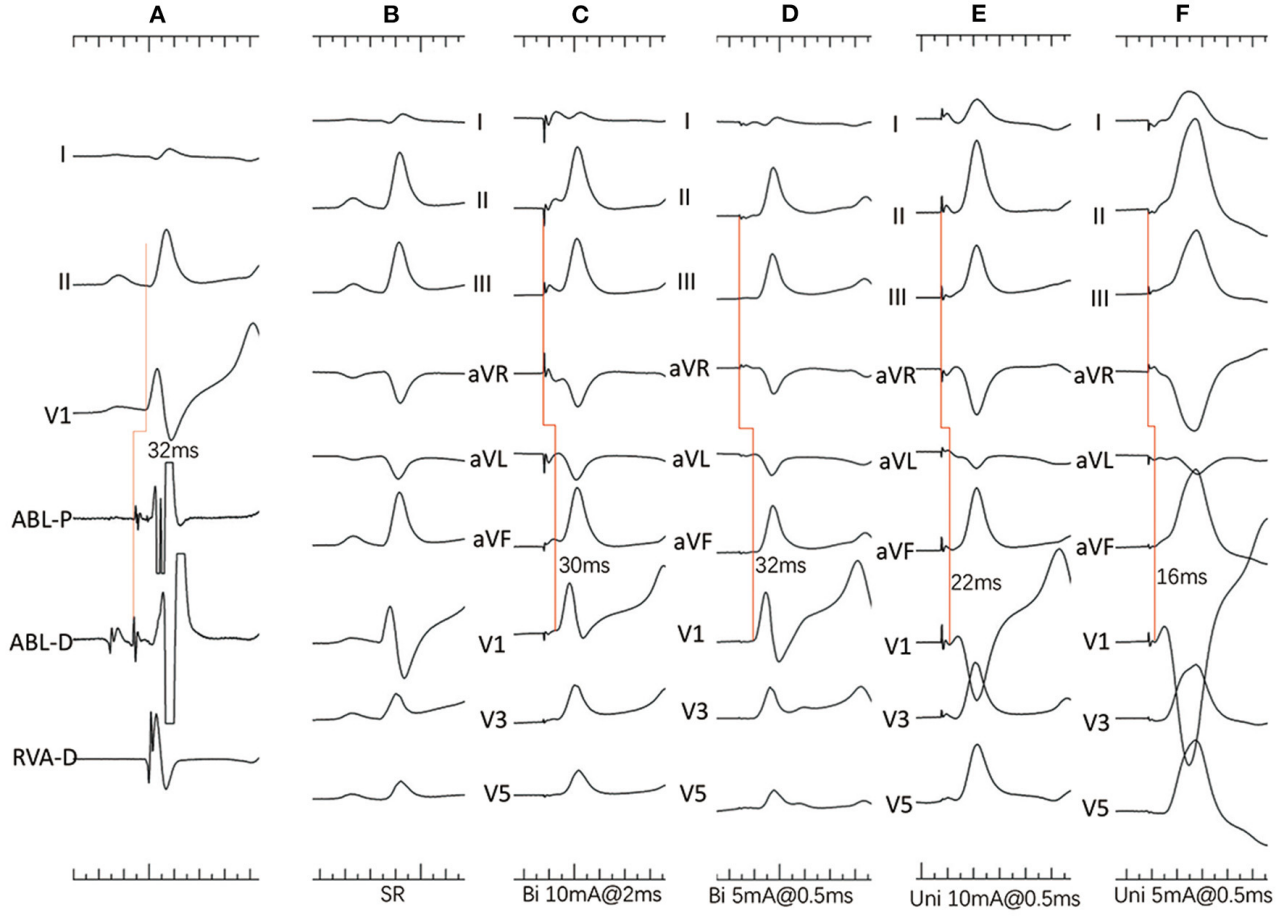
**(A–F)** Dog #2 (group 2). **(A)** Surface ECG leads I, II, V1, and intracardiac recordings from a quadrupolar catheter in the right ventricle and mapping catheter (ABL) at the apex of the NCC-RCC junction. Note that the H-V interval is 30ms with atrial and His activation on distal electrodes. **(B)** Surface 12-lead ECG during sinus rhythm (SR). **(C)** Bipolar pacing with 10 mA, 2 ms shows slightly different morphology from SR with narrow QRS duration, indicating both myocardial and His capture. **(D)** Bipolar pacing with 5 mA, 0.5 ms shows paced QRS morphology identical to that during SR and a long stimulus to QRS interval indicating pure HB capture. **(E)** Unipolar pacing with 10 mA, 0.5 ms shows narrow QRS complexes with short stimulus-to-QRS interval, indicating both myocardial and His capture. **(F)** Unipolar pacing with 5 mA, 0.5 ms shows wide QRS complexes indicating only myocardial capture.

Unipolar and bipolar pacing from the mapping catheter was performed as previously reported (1). The pacing protocol consisted of different pacing currents (20, 10 and 5 mA) at different pulse widths (2, 1 and 0.5 ms). Pacing with a cycle length of 400–450 ms was only performed at the sites with His activation within the LUS below the NCC-RCC junction. The resultant paced QRS morphologies were showed as Figure 2. The following parameters were analyzed on the recording system at the paced sites: (1) the amplitude of atrial and ventricular potentials, and the A/V amplitude ratio; (2) the amplitude of His potential; and (3) the H-QRS, the stimulus-to-QRS and the QRS duration during pacing and SR.

### Radiofrequency Ablation Protocol

RF energy was delivered as previously reported (1). Before ablation, a 5F catheter was positioned in the right ventricle apex for back-up pacing if AV block occurred. In every dog, only a single application of irrigated RF energy was delivered in power-controlled mode with a power of 30 W and a flow rate of 17 mL/min and maintained for 60 seconds.

In this study, two sites with His activation within LUS below the NCC-RCC junction were chosen for a single RF application. The anatomical site was located at the base of the triangle between the hinges of the RCC and NCC in 6 dogs (group 1) and at the apex of that in 6 dogs (group 2). During RF delivery, the PR interval, abnormal ventricular ectopy or rhythm and catheter movement were continuously monitored. An abnormal ventricular ectopy or rhythm was defined as originating from Purkinje system if a distinct Purkinje activation preceded the QRS during RF ablation. AH and HV intervals were repeatedly measured immediately after the procedure in cases of no AV block. Also, the distance between the targeted site in the LV and the NCC-RCC junction was measured on the 3-D map.

For those dogs with third-degree AV block, a single chamber Sigma permanent ventricular pacemaker was implanted, being programmed to VVI mode with a rate of 120 beats per minute.

### Follow-Up

All dogs were kept alive with continuous monitoring for 12–14 days to investigate whether AV and bundle branch (BB) block persisted, and then sacrificed for pathological examination. Standard and V1, V2 and V3 leads of surface ECGs were recorded under anesthesia with intramuscular administration of ketamine hydrochloride (6 mg/kg) and continuous mask ventilation before sacrificing.

### Pathologic Examination

**Figure 3:**
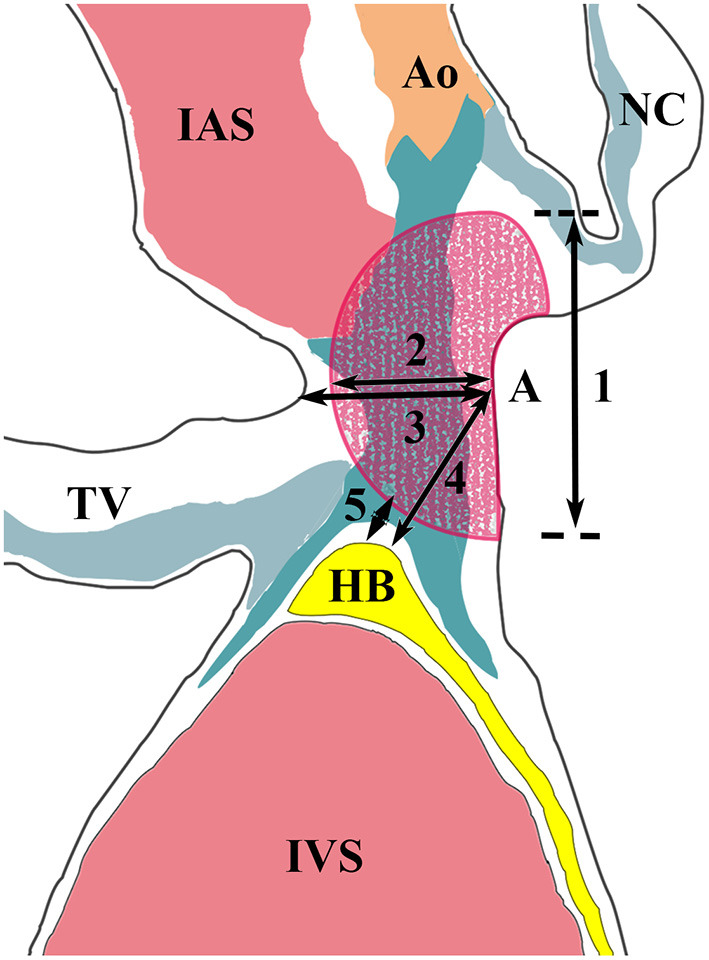
Measurements of the ablation lesion and its related conduction tissue injury. Segments 1 and 2 represent the diameter and depth of the ablation lesion. Segment 3 represents the thickness of the interventricular septum at the ablation site. Segment 4 shows the distance between the ablation site and the His bundle. Segment 5 represents the shortest distance between them. Ao, aorta; HB, His bundle; IAS, interatrial septum; IVS, interventricular septum; NC, non-coronary leaflet; TV, tricuspid valve.

Pathologic examination was performed as previously reported (1). All tissue sections were stained with hematoxylin and eosin and Masson trichrome using routine protocols. The ablation lesion and its related conduction tissue injury were histologically determined at the tissue section with the maximum ablation injury (Figure 3) (3). In this study, the HB was defined as including the penetrating bundle (PB) within the septum and BB, which extends from the PB to the beginning of the junction of the LBB and right BB (RBB).

### Statistical Analysis

Data are expressed as the mean ± SD or medians with minimum and maximum values. For comparison between groups, the Mann-Whitney *U*-test was used for the continuous variables. A two-sided *P* < 0.05 was considered statistically significant. Analyses were performed using IBM SPSS Statistics 22.

## Results

### Basic Electrophysiological and Mapping Data

There was no statistical difference in the baseline AH, HV intervals and QRS duration during SR between the two groups. LV mapping was performed via only the transaortic approach in 6 (#4, #5 and #6 in group 1, #2, #4 and #6 in group 2) and via both transaortic and transseptal approaches in the other 6 dogs. High-density maps in the aortic root and around the LUS below the NCC-RCC junction were successfully achieved with 51 ± 13 (range 33–74) points and 56 ± 25 (range 24–104) points. A small triangular area with His activation was found below the NCC-RCC junction in all 12 canine models. This small triangular area measured 0.950 ± 0.197 cm∧2 (range 0.6–1.2 cm∧2) via an only transaortic approach and 0.933 ± 0.334cm∧2 (range 0.6–1.5cm∧2) via both transaortic and transseptal approaches (*P* = 0.805). The distance between the apex of the triangle and the middle base of the triangle was 8.95 ± 2.32 mm in these 12 dogs. The amplitude of atrial, His and ventricular activation were 0.70 ± 0.50 mV, 0.51 ± 0.35 mV, and 1.41 ± 0.85 mV at the apex of the triangle, and 0.42 ± 0.33 mV, 0.37 ± 0.20 mV, and 4.45 ± 3.70 mV at the middle base of the triangle, respectively. An FF His activation was recorded above the NCC-RCC junction in all 12 dogs, with the amplitude of 0.22 ± 0.12 mV.

### Pacing Protocol at the Apex and Middle Buttom of the Triangle With His Activation

Two sites were chosen for ablation after completing the pacing protocol. One was located at the base of the triangle between the hinges of the RCC and NCC 8.03 ± 1.18 mm away from the apex (group1) and the other was at the apex 3.42 ± 0.61 mm away from the RCC-NCC junction (*P* = 0.004) (Table 1). The His and atrial amplitudes were not significantly different between both groups. However, the amplitude of ventricular activation was significantly larger in group 1 (*P* = 0.01), with a lower A/V ratio (*P* = 0.01). The pacing protocol was performed via transaortic approach in three and transseptal approach in three in each group.

**Table 1:** Eletrophysiologic study, mapping, and ablation data.

	**Gruop1**	**Gruop2**	***P* values**
AH interval (ms)	62 ± 16	53 ± 10	0.15
HV interval (ms)	32 ± 2	33 ± 5	0.93
Wenckenbach cycle (ms)	262 ± 40	242 ± 43	0.47
Local amplitude at targeted site (mV) Atrial activation	0.24 ± 0.13 (0.13–0.48)	0.34 ± 0.26 (0.14–0.79)	0.75
Ventricular activation	2.40 ± 1.76 (0.54–4.39)	0.52 ± 0.26 (0.28–0.99)	0.01
A/V ration	0.15 ± 0.09 (0.07–0.24)	0.79 ± 0.81 (0.22–2.38)	0.01
His activation	0.28 ± 0.19 (0.15–0.65)	0.25 ± 0.15 (0.05–0.44)	1.00
The targeted site to the NCC-RCC apex (mm)	8.03 ± 1.18 9.4) (6.5–9.4)	3.42 ± 0.61 (2.5–4.3)	0.04

*Abbreviations as in Figure 1*.

In group one, the minimal output required to capture the HB with bipolar pacing was 5 mA/0.5 ms in all six dogs, and with unipolar pacing was 5 mA/0.5 ms in three dogs and 10 mA/0.5 ms in the remaining 3 dogs. Bipolar paced QRS duration and Stimulus-to-QRS intervals were 96.00 ± 7.48 ms and 32.00 ± 2.83 ms, whereas unipolar paced QRS duration and Stimulus-to-QRS intervals were 96.80 ± 5.22 ms and 28.80 ± 8.67 ms. In dog #3, minimal output during unipolar pacing resulted in simultaneous atrial capture. Pure HB capture was observed in five with minimal bipolar pacing output and in one dog with minimal unipolar pacing output.

In group 2, the minimal output required to capture the HB with bipolar pacing was 5 mA/0.5 ms in 3, 10 mA/0.5 ms in 1, 10 mA/1 ms in 1 and 10 mA/2 ms in one dog. Whereas the minimal output required to capture the HB with unipolar pacing was 10 mA/0.5 ms in 3, 10 mA/1 ms in 2 and 10 mA/2 ms in 1 dog. Bipolar paced QRS duration and Stimulus-to-QRS intervals were 92.67 ± 12.24 ms and 30.33 ± 4.46 ms. Unipolar paced QRS duration and Stimulus-to-QRS intervals were 100.67 ± 15.47 ms and 26.00 ± 7.48 ms. Pure HB capture was observed in 3 dogs with bipolar pacing and none with unipolar pacing.

There was no significant difference in paced output between bipolar and unipolar pacing in both groups. Also, the paced QRS duration and Stimulus-to-QRS interval in both groups were not significantly different with bipolar or unipolar pacing.

### Irrigated RF Ablation

**Figure 4:**
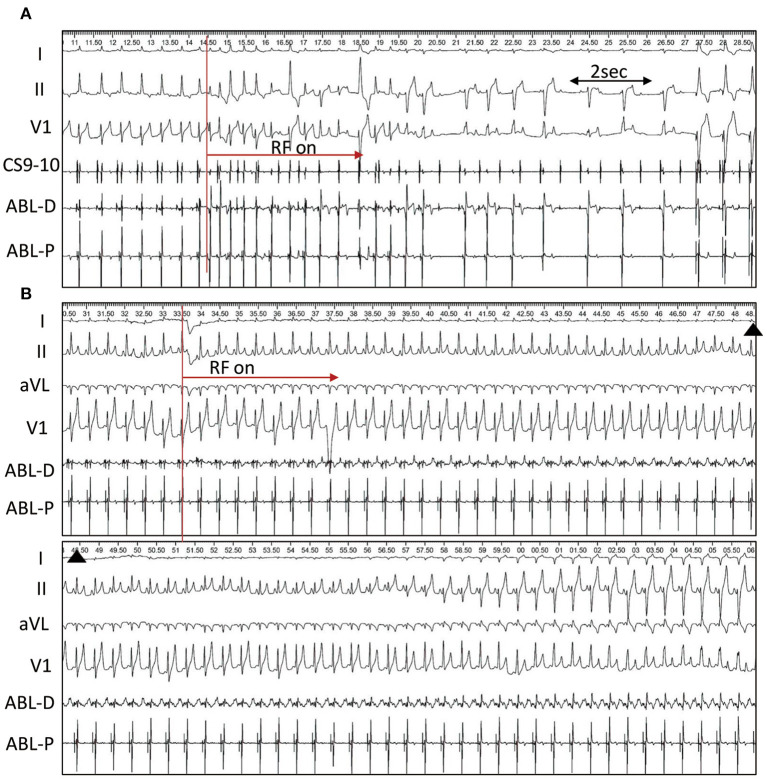
Single RF application at the triangle. **(A)** Radiofrequency (RF) Ablation in dog #4 (group 1). RF energy delivery results in immediate alternating narrow and wide QRS junctional beats, and complete AVB occurs at 4.4s after ablation followed by ventricular paced rhythm. **(B)**, Ablation in dog #2 (group 2). Right bundle branch block and left anterior fascicular block occurs 28.8s after ablation and persists until the end of the procedure.

In group 1, a single RF application was delivered at the middle base of the triangle interposed between the hinges of the RCC and NCC. RF energy resulted in immediate Purkinje-associated ventricular rhythm, with a narrow QRS morphology in one, wide QRS morphology with RBB block(RBBB) and superior axis in three and alternating narrow and wide QRS morphology in 2 dogs. III degree AV block was observed after cessation of the junctional rhythm in all 6 dogs (Figure 4A). A slow junctional escape rhythm with cycle lengths of 1,358 ± 537 ms (616–2096 ms) was recorded after the ablation. A VVI pacemaker with HR set at 120 bpm was immediately implanted via the right jugular vein.

In group 2, a single RF application was delivered at the apex of the triangle. PR prolongation and AV block was not observed during the single RF ablation. However, Purkinje-associated junctional beats or salvos were infrequently observed in 3 dogs. In dog #2, RBBB and left anterior fascicle block developed 28.8s after ablation via a transaortic approach and persisted until the end of the procedure (Figure 4B). In dog #5, via a transseptal approach, the RBBB and left anterior fascicle block were seen 47.4s following the start of ablation, and completely recovered 4.0s after stopping the RF application. At the end of the procedure, HV intervals were repeatedly measured and remained unchanged from baseline in all 6 dogs.

### Follow-Up

In group 1, dog #1 had undergone pacemaker implantation and died suddenly 1 day after ablation. Pathological examination found a myxoma on each side of the interatrial septum near the inferior margin of the fossa ovalis. The right myxoma was ruptured with a short stump at the fossa ovalis. The long section (56 mm length, 4–10 mm width) had embolized into the right ventricular outflow tract. It was most likely due to the transseptal approach. The left-sided myxoma (50 mm length, 4–10mm width) remained intact. In the other 5 dogs, ECG recordings showed persistent paced rhythm with heart rate of 120 beats/min and AV dissociation.

In group 2, PR prolongation and AV block was not observed in any dogs. In dog #2, RBBB with left anterior fascicle block remained even on Day 14 post ablation.

### Pathologic Findings

Pathological examination was performed 12–14 days after ablation in both groups except dog #1 from group 1, who underwent pathological assessment 1 day after the ablation. Focal hemorrhage was found only in the tricuspid valve in 2/5 dogs in group 1 and 1/6 in group 2. Infective endocarditis was found on the tricuspid valve and left ventricular outflow tract in 1/5 from group 1 and 1/6 from group 2.

**Figure 5:**
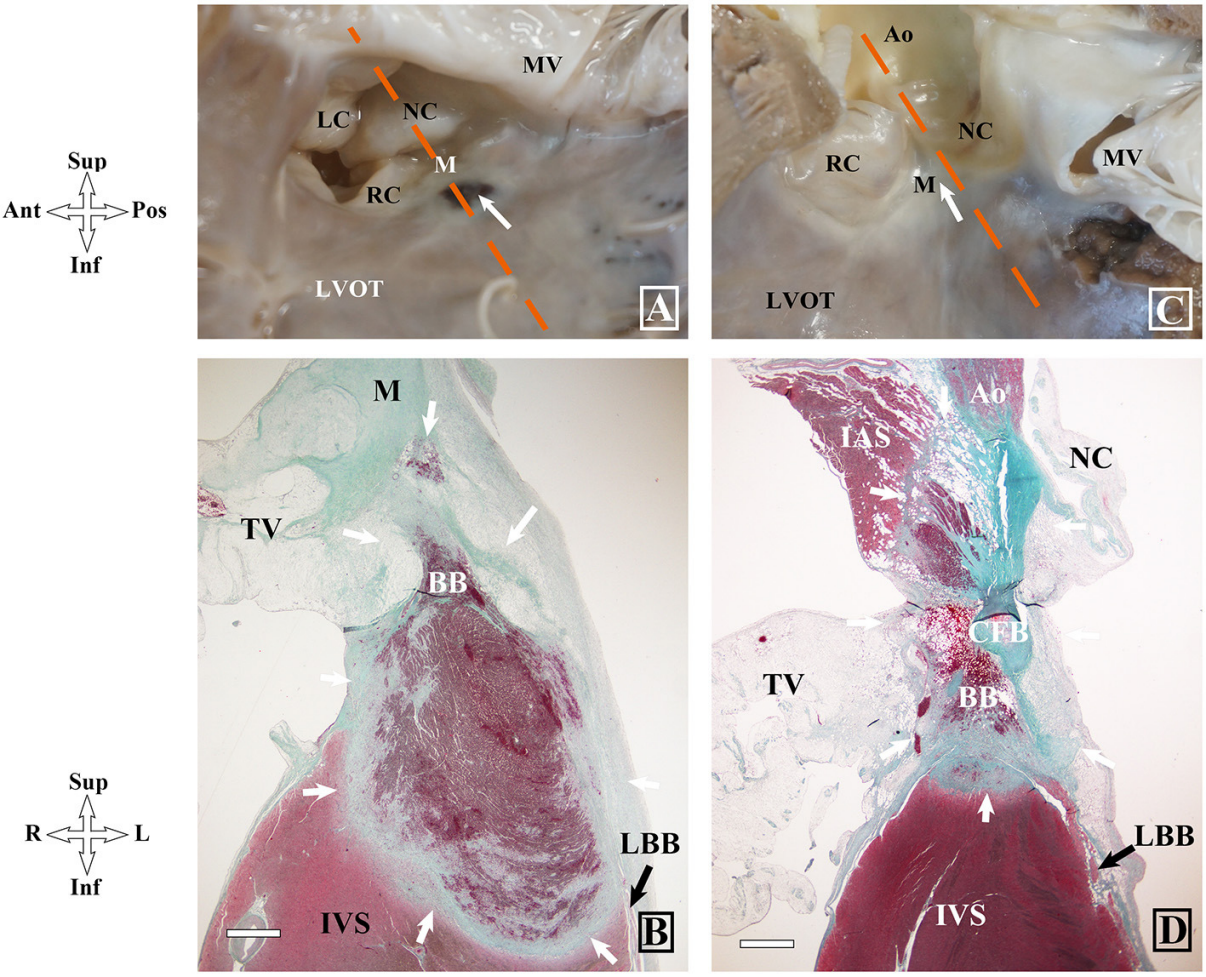
Gross and histopathologic images of different locations of ablation lesions and related injuries on atrioventricular conduction system in dog hearts after radiofrequency delivery. **(A,B)** Coagulative necrosis is shown at the left upper septum below the IMS in dog #5 (group1). **(C,D)** Complete necrosis in the IMS in dog #2 (group2). BB, bundle branch; IMS, interventricular membranous septum; LAF, left anterior fascicle; LBB, left bundle branch; PB, penetrating bundle; RBB, right bundle branch; RC, right coronary leaflet; LC, left coronary leaflet.

In group 1 where all dogs had persistent AV block (all at the infra nodal site), coagulative necrosis occurred just below the interventricular membranous septum (IMS) and extended into the PB,BB and LBB in dogs #3–#6 (Figures 5A,B). In dog #1, coagulative necrosis occurred infero-posterior to the IMS extended inferiorly and anteriorly and resulted in complete necrosis of the PB/BB and LBB and in dog #2, the lesion occurred in the IMS leading to complete necrosis of the PB/BB and incomplete necrosis of the LBB. However, there was no injury in the AVN and RBB in all 6 dogs of this group.

In group 2, coagulative necrosis was not found in dogs #4 and #6 with a transaortic approach. Coagulative necrosis was found at the postero-superior region of the IMS without any injury of the AVCS in dog #1. Coagulative necrosis was found below the NCC and extended anteriorly and superiorly, which resulted in complete necrosis of the RBB within the septum, and complete injury of the proximal left anterior fascicle, in dog #2 with persistent RBBB and left anterior fascicle block after ablation (Figures 5C,D). In dog #5 with transient RBBB and left anterior fascicle block, coagulative necrosis was found at the IMS and extended to the upper margin of the RBB and resulted in partial necrosis of the RBB without involvement of the LBB.

There was no difference in the length of IMS (3.6 ± 0.6 vs. 3.3 ± 0.4; *P* = 0.292), the PB (5.22 ± 1.05 mm vs. 5.07 ± 1.57 mm; *P* = 0.463), the BB (5.33 ± 0.71 mm vs. 5.70 ± 1.13 mm; *P* = 0.747) and ablation lesion width (6.55 ± 1.17 mm vs. 5.08 ± 1.38 mm; *P* = 0.109) between the groups. However, the lesions in group 1 were markedly deeper than that in group 2 (5.20 ± 2.43 mm vs. 2.40 ± 0.87 mm; *P* = 0.025), which may be due to thicker tissue at the ablation sites in group 1 (5.10 ± 1.75 mm vs. 3.44 ± 1.15 mm; *P* = 0.078). In group 1, RF-related complete AV block demonstrated different levels of PB/BB necrosis.

### Left Para-Hisian Accessory Pathway

**Figure 6:**
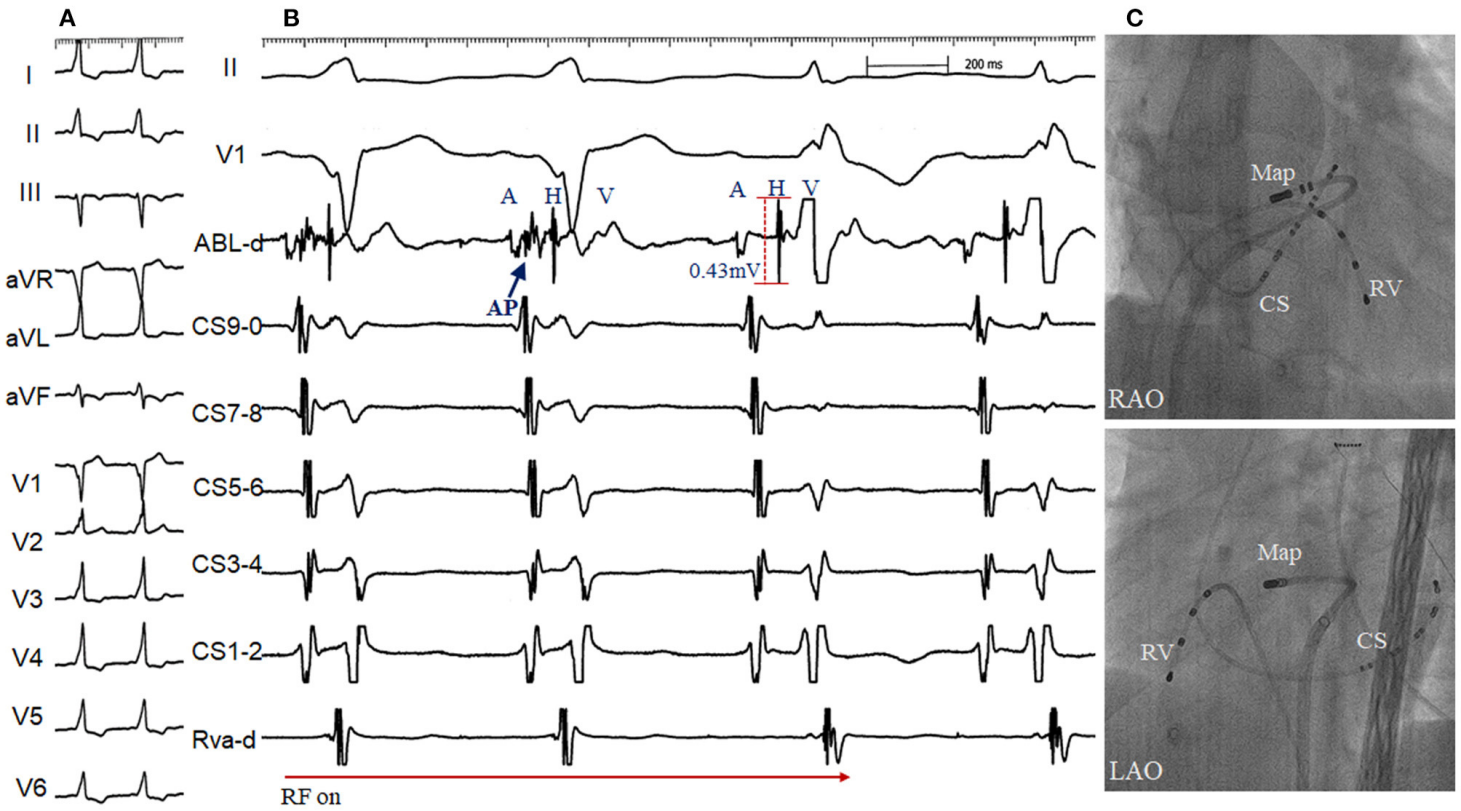
Radiofrequency (RF) delivery **(A)** and fluoroscopic image **(C)** at the apex of the interventricular membranous septum via transseptal approach in a patient with supra-paraseptal ventricular preexcitation **(B)** and 2 failed RF attempts at the right para-Hisian region and non-coronary cusp (Right bundle branch block developed after the second ablation). The accessory pathway blocked within 2s during the first RF application, starting with 15W and up-titrated to 30 W. Note a distinct accessory pathway (AP) and large His activation (amplitude 0.43 mV) was recorded at the successful site. ABL, ablation catheter; RV, right ventricle. Abbreviations as in Figure 1.

A 32-year-old male presented with recurrent paroxysmal supraventricular tachycardia. Twelve-lead ECG in SR showed supra-paraseptal ventricular preexcitation. A transthoracic echocardiogram was normal. Two previous ablations at the right PH region and NCC failed to eliminate the antegrade and retrograde conduction of the accessory pathway, and RBBB developed after the second ablation. At the third procedure, the accessory pathway with a large His activation was successfully eliminated by ablation via a transseptal approach (Figure 6). Angiography of the aorta confirmed the position of catheter located at the apex of the IMS. No junctional beats occurred during ablation and the HV interval remained stable to that during tachycardia. No preexcitation on surface ECG or evidence of tachycardia-related symptoms was documented off antiarrhythmic drugs during a follow-up of 30 months.

## Discussion

This study describes the methods to identify the anatomical distribution of the left HB, and assesses the clinical-pathological correlation when RF application is targeted at LUS below the NCL-RCL junction.

### Anatomical Distribution of the Left His Activation on 3-D Mapping

The LUS leans to the aortic valves. It is here the common atrioventricular conduction bundle emerges from the central fibrous body to pass along the base of the membranous septum and the crest of the muscular ventricular septum (4). In both the human and canine hearts, the atrioventricular node is uniformly located at the apex of the inferior pyramidal space. It is the presence or absence of the inferoseptal recess, therefore, that is the major difference between the two species. In turn, in canine and the minority of human hearts, a more extensive nonbranching bundle skirts half of the noncoronary aortic sinus (5). Our pathologic results showed that the length of IMS in canine was shorter than that in human heart, according closely with previous study (5, 6). The landmark for the site of the atrioventricular conduction bundle is the central fibrous body that adjoins the interleaflet triangle interposed between the hinges of RCL-NCL. From here, the LBB descends in the subendocardium and usually branches into three main fascicles.

Given the thin HB, the HB potential should not be recorded in a large area as frequently shown in 3D mapping. In this study, a triangular area (0.9 cm∧2) with a discrete His potential was found below the RCL-NCL junction using high-density mapping, via both the transaortic and transseptal approach. The anatomical distribution of the His activation was inconsistent with the anatomical assessment that the left His runs along the septal crest. The most possible explanation is FF vs. NF His activation at this area.

### Identification of Near-Field and Far-Field His Activation in the Left Ventricle

Our previous studies have demonstrated that NF or FF His activation can be identified using systematic pacing maneuvers in the right PH region (1, 2). However, in this study, unipolar and bipolar pacing both failed to discern NF and FF His activation in the left PH region. There was some overlap between group 1 and group 2 in the minimal output required to capture NF His activation. In addition, de and colleagues' histological study have shown that the nonbranching bundle are significantly longer in dog than in man (5). The great proportion of the AV conduction axis skirts the noncoronary sinus of the aortic root within the right atrial vestibule. According to the above-mentioned results, we can speculate that the pacing protocol used in our study might unable distinguish NF and FF His activation in the left PH region in dog. Due to the inability to differentiate NF from FF His activation at the triangle, we hypothesized that the His activation at the apex of the triangle was FF activation and NF was located at the base of the triangle. Then, similar to our previous study in the right PH region, a single RF energy was attempted to delivery at the two sites with His activation for confirming NF and FF His activation.

In this study, patho-histologic assessment 12–14 days after ablation showed clear coagulative necrosis with a diameter of 5–7 mm, consistent with previous animal studies. In the 6 dogs from group 1, RF-induced coagulative necrosis of the PB/BB and LBB in 5 dogs and complete necrosis of the PB/BB and incomplete necrosis of the LBB in 1, which resulted in total AV block and was consistent with the findings on the surface ECG. In group 2, RF resulted in no AV block in all 6 dogs. No complete coagulative necrosis occurred at the PB and proximal BB except in one dog, which was proved without changed HV interval before and after ablation. The mechanism of damage to the RBB and left anterior fascicle may be associated with direct thermal effect or lesion expansion generated by RF energy ablation as well as slight catheter dislocation during ablation. On the other contrary, no evidence of RF-induced lesion with an energy of 30 w was found in 2 dogs. However, the diameter of coagulative necrosis in the remaining 4 dogs was similar to that in the 6 dogs of group 1. The above results indicated that poor contact of ablation catheter with transaortic approach may be related with no RF-induced lesion.

Compared with group 1, we found a larger atrial electrogram and higher A/V ratios vs. group 2 compared to these in groups. It can be explained by anatomical findings that the apex of the triangle is closer to the atrium and the base is closer to interventricular myocardium.

### Clinical Implication of Catheter Ablation Close to the Left His Bundle Region

The critical concern of ablation close to the left HB region is durable injury of the AVCS, and AV conduction disturbances requiring permanent pacemaker implantation are not uncommon (7). In addition, the risk of severe conduction abnormalities can be influenced by anatomical variation of the conduction system and baseline disturbances. Our observation highlights a potential way to minimize AV injury during ablation close to the triangle between the hinges of NCL-RCL. The first step is to identify the anatomical location of the triangle, which is completely covered with NF-and FF-His activations. The atrial amplitude and AV ratio can also indirectly help identify the anatomical location. The second step is to initially deliver RF with low energy which can be up-titrated at the apex when it is necessary to ablate these arrythmias. The AV interval and junctional response should be continuously monitored.

Our case with a patient with the left PH AP further demonstrated our ablation strategy that RF application with low energy can be safely delivered at the apex of the triangle in spite of a large His activation (0.43 mV). On the other hand, no change on surface ECG and HV interval does not mean no pathological injury of the left-HB system. Finally. it needs further study whether our method can also be extrapolated into mechanical or transcatheter aortic valve replacement procedure to minimize or avoid AV block.

### Study Limitations

In the study, a steerable 7.5F, D-curve catheter with a 3.5-mm irrigated-tip electrode was used for mapping and pacing in in the study. No high-resulation catheter such as PentaRay, Orion catheter were used. However, it may be very difficult to reach the region in small hearts. Secondly, the AVCS in dogs differs from the that in humans. It need further investigation whether our experimental data can be extratrasplated into clinical practice in humans, especially in the elderly patients. Finally, because intracardiac echocardiography was not used to identify the valvar leaflet in aortic root, we used “cusp” to show the catheter location in the method and results.

## Conclusion

The His activation can be widely distributed in the LUS below the RCL-NCL junction. It was difficult to differentiate the NF-and FF-His activation using pacing technique at the region. However, anatomical location of His activation in the triangle between the hinges of RCL-NCL can help identify the NF- from FF-His activation, which was consistent with clinical-pathological finding with a single RF application at apex or base of the triangle of that in canine model. This important information can provide an objective evidence to guide catheter ablation in arrhythmias originating from in LPH area.

## Data Availability Statement

The original contributions presented in the study are included in the article/supplementary material, further inquiries can be directed to the corresponding authors.

## Ethics Statement

The studies involving human participants were reviewed and approved by Ethic Committee of Wuhan Asian Heart Hospital. The patients/participants provided their written informed consent to participate in this study. The animal study was reviewed and approved by the Institutional Animal Care and Use Committee at Guangdong Provincial People's Hospital. Written informed consent was obtained from the individual(s) for the publication of any potentially identifiable images or data included in this article.

## Author Contributions

All authors listed have made a substantial, direct, and intellectual contribution to the work and approved it for publication.

## Funding

The study was partially supported by Science and Technology Program of Guangdong Province, China (No. 2014B070705005 to SW), National Key Research and Development Project (No. 2018YFC1312502), and Science and Technology Planning Program of Guangdong Province, China (No. 2019B020230004 to SW).

## Conflict of Interest

The authors declare that the research was conducted in the absence of any commercial or financial relationships that could be construed as a potential conflict of interest.

## Publisher's Note

All claims expressed in this article are solely those of the authors and do not necessarily represent those of their affiliated organizations, or those of the publisher, the editors and the reviewers. Any product that may be evaluated in this article, or claim that may be made by its manufacturer, is not guaranteed or endorsed by the publisher.

## Abbreviations

AV: atrioventricular
AVCS: AV conduction system
BB: bundle branch
CS: coronary sinus
FF: far-field
IMS: interventricular membranous septum
LBB: left bundle branch
LCC: left coronary sinuses
LUS: left upper septum
LV: left ventricle
NCC: noncornary coronary cusp
NCL: noncornary coronary leaflet
NF: near-field
PB: penetrating bundle
RBB: right BB
RBBB: RBB block
RCC: right coronary cusp
RCL: right coronary leaflet
RF: radiofrequency
SR: sinus rhythm.
